# Long‐Term Control of Human Papillomavirus‐Related Focal Epithelial Hyperplasia in an Human Immunodeficiency Virus‐Positive Patient Using Methylene Blue‐Mediated Photodynamic Therapy. A Case Report

**DOI:** 10.1002/lsm.70091

**Published:** 2025-12-27

**Authors:** Juliana Cristina Oliverio de Araújo, Hermano Camelo Paiva, Paula Margarida Meyer Faara, Og Fray Junior, Martha Simões Ribeiro

**Affiliations:** ^1^ Professional Master Radiation Technology in Health Sciences Nuclear and Energy Research Institute (IPEN‐CNEN) São Paulo Brazil; ^2^ Dental Specialties Center “Brasil Sorridente” Osasco Brazil; ^3^ Center for Lasers and Applications Nuclear and Energy Research Institute (IPEN‐CNEN) São Paulo Brazil; ^4^ Department of Restorative Dentistry, School of Dentistry University of São Paulo São Paulo Brazil

**Keywords:** FEH, Heck's disease, immunodeficiency, MB, oral diseases, oral warts, PDT, red laser irradiation

## Abstract

**Background:**

Human papillomavirus (HPV) infections are a major cause of oral lesions, and in individuals living with HIV, lesions such as focal epithelial hyperplasia (FEH) may persist or exhibit atypical features, potentially progressing to more severe conditions if untreated. Managing oral HPV lesions in immunocompromised patients is challenging, as conventional therapies may carry higher risks or show limited efficacy.

**Methods:**

This study reports the case of a 49‐year‐old HIV‐positive male with valve disease and arthritis, requiring crutches for mobility. He presented with multiple painless oral lesions, diagnosed as FEH associated with oral HPV, and had previously undergone unsuccessful treatments. Photodynamic therapy (PDT) using methylene blue (MB) and a red laser was proposed as a treatment.

**Results:**

Topical MB‐mediated PDT successfully cleared the FEH lesions, with no recurrence observed over 24 months.

**Conclusion:**

PDT mediated by MB is an effective and affordable approach for treating FEH associated with HPV in immunosuppressed patients, offering favorable outcomes and improved quality of life.

## 1 Introduction

Human papillomavirus (HPV) is a small DNA virus that has a specific tropism for squamous epithelia. To date, 231 different clones of HPV have been identified [1]. The types of HPV that infect mucous membranes are classified into high and low risk groups, depending on the malignant potential of the lesions they cause. Low‐risk HPVs, such as HPV6 and HPV11, are associated with benign warts, while high‐risk HPVs, such as HPV16 and HPV18, can cause premalignant squamous intraepithelial neoplasia, which has the capacity to develop into cancer [2].

Human immunodeficiency virus (HIV)‐positive individuals have a higher prevalence of oral HPV infections compared to immunocompetent individuals. However, the introduction of highly active antiretroviral therapy (HAART) in the treatment of HIV‐positive people has been associated with an increase in the occurrence of focal epithelial hyperplasia (FEH) [3]. Indeed, HPV‐associated oral warts, including FEH, have a prevalence of 0.5% in the general population but occur in up to 5% of HIV‐seropositive individuals and up to 23% of those receiving HAART [3]. While antiretroviral treatment remains the most effective approach for long‐term viral suppression, reducing both morbidity and mortality, it does not eradicate HIV infection, necessitating lifelong treatment in most cases [4].

However, despite the benefits of HAART, HPV‐related complications remain a significant concern in individuals with HIV. First‐line treatments for HPV‐associated lesions include surgical excision, cryotherapy, electrocoagulation, and carbon dioxide laser therapy but these approaches are invasive and may be associated with relevant risks and side effects [5]. Although life expectancy in this population has markedly improved, the incidence of HPV‐related head and neck squamous cell carcinoma is expected to increase in the coming years [6]. Additionally, as individuals with HIV age, they are more likely to develop chronic comorbidities such as cardiovascular disease, liver disease, and diabetes mellitus [7], which can further complicate the management of HPV‐related lesions. These conditions increase the risk of bleeding, delayed wound healing, and postoperative infections, making surgical interventions less suitable for some patients. In this context, minimally invasive therapeutic alternatives represent particularly valuable treatment options.

Photodynamic therapy (PDT) is a non‐invasive therapeutic approach that has have shown promising results to treat warts [8]. This technique combines a photoactivatable dye, called photosensitizer (PS), which, when exposed to light of an appropriate wavelength, enters an excited state and generates reactive oxygen species [9]. PDT provides dual selectivity by targeting cells through both light activation and the preferential accumulation of the PS.

A widely used PS is methylene blue (MB). This compound, which has been developed in the 19th century, has proven to be effective under red light to reduce a broad range of microorganisms, including Gram‐positive and Gram‐negative bacteria, fungi, protozoa, and viruses [10, 11]. Indeed, MB has the potential to treat a variety of diseases, both cancerous and non‐cancerous, with low toxicity and no significant side effects. Documented clinical cases include treatments for basal cell carcinoma, Kaposi's sarcoma, melanoma, cutaneous leishmaniasis, and viral and fungal infections [12].

## 2 Case Report

In May 2023, a 49‐year‐old HIV‐positive male patient, with a history of valve disease, including an aortic mechanical prosthesis and mitral valve repair, as well as arthritis and reliance on crutches, was referred to the Dental Specialties Center (CEO) in Osasco, São Paulo, Brazil, by an infectious disease specialist for dental treatment. He reported beginning treatment at Emílio Ribas Hospital 2 years earlier and was on a regimen of prednisolone, carvedilol, enalapril, furosemide, simvastatin, darunavir, ritonavir, dolutegravir, lamivudine, and sulfamethoxazole.

During anamnesis, his primary concern was the presence of multiple painless oral lesions that had persisted for several years, affecting both esthetics and eating, despite previous surgical removal.

**Figure 1:**
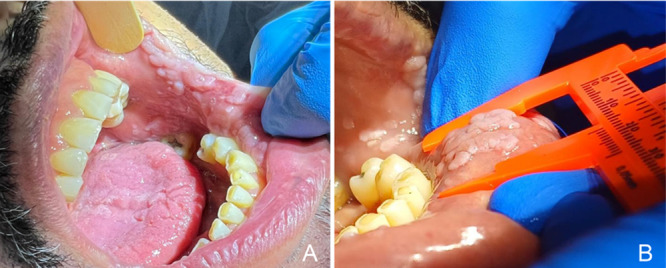
Clinical appearance of lesions on the buccal mucosa (A) and lower labial mucosa (B) in May 2023, prior to treatment initiation. The average lesion size was approximately 5 mm, although areas exceeding 4 cm^2^ were observed.

Clinical examination revealed multiple sessile, raised lesions affecting the upper and lower lips, buccal mucosa, dorsum of the tongue, alveolar ridge, and palate (Figure 1). Excisional biopsies analyzed by a specialized laboratory confirmed FEH associated with oral HPV. Chromogenic *in situ* hybridization was inconclusive for both high‐risk and low‐risk HPV DNA.

After the patient provided written informed consent to participate in the study, we proposed PDT, initially using a 1% MB solution injected intralesionally into the affected areas. Due to the patient's medical condition, 2 g of amoxicillin was administered 1 h before the procedure as antibiotic prophylaxis. After a 10‐min incubation period for MB uptake, all visible oral lesions were irradiated point by point, corresponding to the spot area, using a red laser (660 nm, MMOptics, São Carlos, SP, Brazil) with an output power of 100 mW and a spot area of 3 mm², delivering 9 J per irradiation point over 90 s. Lesions larger than 4 cm² received irradiation at two or more points to ensure complete coverage. PDT was performed in five sessions on alternate days. The patient reported no pain, discomfort, or adverse effects following PDT and stated that the transient blue discoloration resolved by the following day.

**Figure 2:**
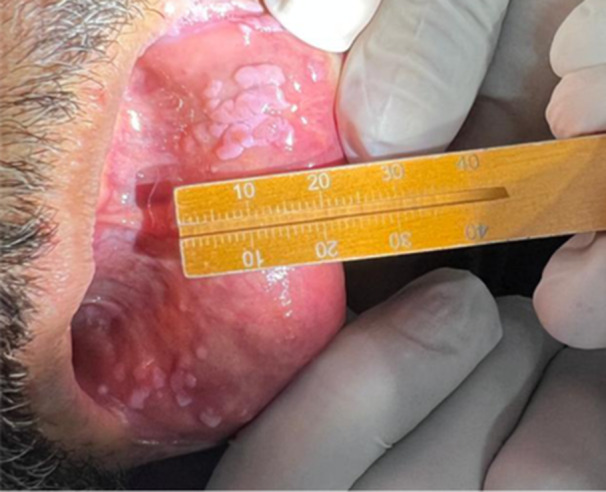
Clinical appearance of the lesions 30 days after the final session of MB‐mediated PDT and before initiation of the second PDT series.

**Figure 3:**
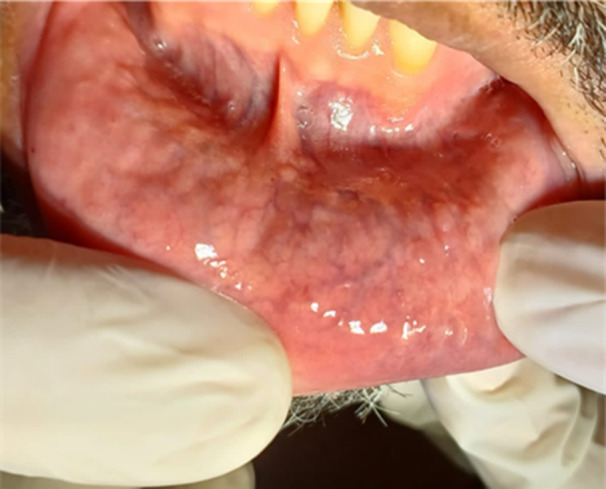
Clinical appearance of the lower labial mucosa 5 months after the final session of the second MB‐mediated PDT series and before preventive treatment.

After 30 days, the patient returned for follow‐up assessment, which revealed lesion flattening without complete resolution (Figure 2). To avoid the need for antibiotic prophylaxis, we proceeded with five additional PDT sessions using topical application of MB. A gauze soaked in MB was applied to the lesions for 10 min, followed by irradiation with the red laser, maintaining the same light parameters. The patient was scheduled for re‐evaluation 30 days after, but due to health issues, returned 5 months later, at which time no lesions were observed (Figure 3).

**Figure 4:**
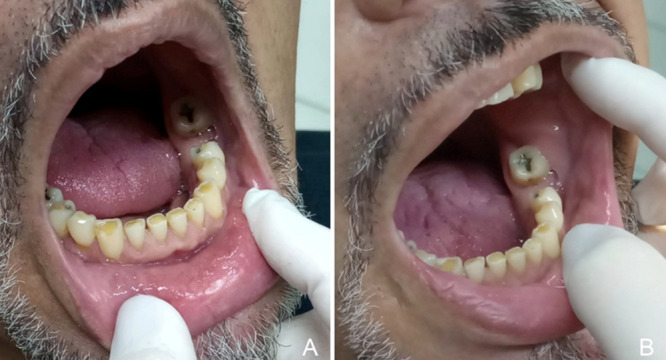
Clinical appearance of the lower labial mucosa (A) and buccal mucosa (B) 24 months after the last treatment session.

The patient has been followed for 24 months and currently attends monthly follow‐up visits, when possible, for recurrence monitoring (Figure 4). PDT with topically applied MB is still performed. However, as no clinically apparent lesions remain, irradiation is now delivered using a scanning technique rather than point‐by‐point application. After a 10‐min MB incubation period, the affected region is irradiated for 2 min, delivering 12 J to the treated area.

## 3 Discussion

Here, we report for the first time the use of MB‐mediated PDT to manage FEH in an HIV‐positive individual, a condition that is often difficult to treat, particularly in the presence of comorbidities. One of the key advantages of PDT is its minimally invasive nature, offering effective treatment with less pain and discomfort than traditional surgical methods. This is especially beneficial for patients who need to avoid aggressive interventions.

PDT relies on a photoactive molecule, here, MB, which selectively accumulates in target cells and induces oxidative damage upon light activation. To maximize its effectiveness, MB must be applied to the lesions for a specific period before irradiation (10 min in our approach). Additionally, the light exposure must be carefully controlled to deliver the necessary energy for optimal therapeutic outcomes (9 J per point in this case).

Initially, five sessions of intralesional MB application were performed, leading to a reduction in lesion size. However, the lesions persisted, prompting a shift to topical MB application. We hypothesize that intralesional MB injections may have further impaired the healing process, possibly due to injection trauma. Furthermore, topical application eliminates the need for antibiotic prophylaxis, simplifying the treatment protocol.

Although the patient returned 5 months after the second series of five PDT sessions, he reported significant improvement in the lesions within 15 days, suggesting that topical MB effectively penetrated the lesions and induced oxidative stress upon red laser irradiation. While PDT has showed lower recurrence rates in the treatment of warts [13], no studies have evaluated its effectiveness in preventing recurrence in individuals living with HIV, who are at a higher risk of persistent HPV infections. Therefore, we continued MB‐mediated PDT with technique refinements, such as employing scanning motions and delivering a higher energy dose (12 J over 2 min) across the entire surface in the absence of visible lesions, to prevent recurrence. No relapses were observed over a 24‐month follow‐up. Extending exposure time and scanning the entire area ensures more uniform energy distribution, enhancing light penetration across all potential sites of infection and reducing the risk of untreated regions that could lead to recurrence.

While PDT mediated by 5‐aminolevulinic acid and methyl aminolevulinate (both precursors of protoporphyrin IX, the PS) has been shown to effectively treat FEH [14] and HPV‐related lesions in an immunosuppressed individual [15], MB‐mediated PDT represents a more affordable and promising alternative. This approach shortens the required dark incubation period, improving patient compliance, and can be also applied to various oral cancers and infectious lesions within the oral cavity, making it a practical and broadly applicable therapeutic option for different oral diseases [16].

In conclusion, topical MB‐mediated PDT successfully managed HPV‐related FEH in an immunocompromised individual with history of valve disease. This outcome supports the need for further validation through clinical trials.

## Funding

The authors received no specific funding for this work.

## Ethics Statement

The patient provided written informed consent for participation in this work.

## Conflicts of Interest

The authors declare no conflicts of interest.
